# Molecular subtyping of *Treponema pallidum* and associated factors of serofast status in early syphilis patients: Identified novel genotype and cytokine marker

**DOI:** 10.1371/journal.pone.0175477

**Published:** 2017-04-14

**Authors:** Rui-Li Zhang, Qian-Qiu Wang, Jin-Ping Zhang, Li-Jia Yang

**Affiliations:** 1 Department of Dermatology, Wuxi Second Hospital, Nanjing Medical University, Wuxi, China; 2 National Center for STD Control and Prevention, China Centers for Diseases Control and Prevention, Institute of Dermatology, Chinese Academy of Medical Sciences, Nanjing, China; University of Kentucky College of Medicine, UNITED STATES

## Abstract

Serofast, a persistent nontreponemal serological response observed in early syphilis patients after conventional treatment, remains a concern of clinicians and syphilis patients. No consensus has been established, however, that defines an effective treatment strategy and clarifies the pathogenesis. In this study, 517 patients with early syphilis were enrolled and treated. Twelve months after treatment, 79.3% (410/517) of patients achieved serological cure, 20.1% (104/517) were serofast, and 0.6% (3/517) were serological failures. Multivariate analysis demonstrated that older age (>*40 years*) and lower baseline RPR titer (**≤** 1:8) were associated with serofast status. We also identified 21 *T*. *pallidum* molecular subtypes among early syphilis patients and detected a new subtype, 14i/a. We found that the proportion of 14i/a type in serofast patients was significantly higher than that in patients with serological cure, predicting an increasing risk of serofast status. Levels of chemerin were higher in the serum of serofast cases than serological cure cases, potentially indicating a novel cytokine marker for serofast in early syphilis patients after therapy. We hope that these results contribute to improve guidelines for the management of syphilis patients who experience serofast.

## Introduction

Syphilis remains a worldwide infection caused by the spirochete *Treponema pallidum*. WHO estimated that around 12 million people were actively infected with syphilis every year worldwide [1], despite this ancient disease has been treated by penicillin for 60 years [2] and no *T*. *pallidum* strains resisting penicillin have been reported, the epidemic of syphilis continues to grow in China [3–4]. In 2015 alone, 455,818 new syphilis cases were reported in China. Moreover, the persistent positive serological status of syphilis patients after treatment with penicillin brings about a new challenge to the clinical management of the infection [5–6].

In 1993, consistent with U.S. treatment guidelines, the Centers for Disease Control and Prevention (CDC) of China recommend serological monitoring of syphilis patients after treatment. Under these recommendations, a 4-fold or more declines in nontreponemal test titers indicated an appropriate serological response. However, after treatment, some patients failed to exhibit a serological response, remaining a persistent positive serological reaction [7–8]. This condition is referred to as “serofast state”, defined by persistently low nontreponemal test titers at 12 months after treatment in patients with early syphilis [9–10].

In China, 13.8% for primary syphilis and 35.0% for secondary syphilis were reported to exhibit a serofast status after treatment [11]. Unfortunately, there is still little data explaining the occurrence of the persistent positive serological response. A few recent studies analyzed factors potentially associated with serofast, including the use of therapeutic drug and innate immune response [12–14]. They demonstrated azithromycin treatment and HIV infection might predicted serofast. A latest meta-analyses suggested the age, stage of infection, and baseline titers were the risk factor for serofast as well [15]. However, it is difficult to evaluate the long-term outcomes of patients remaining as serofast state after anti-syphilis therapy.

Chemerin is originally described as a retinoid-responsive gene from skin lesions of psoriatic patients in 2007 [16]. Recent researches had illustrated that chemerin played an important role in a range of inflammatory processes by regulating the chemotactic effects in the immune cells. In addition, the serum levels of chemerin were determined and were demonstrated upregulating in various pathological conditions, including infectious and metabolic diseases [17]. Chemerin is expected to be a marker for several phases of inflammation.

Given that the data of serofast status and predict factors are still lacking, our goal was to evaluate correlates associated with serofast status in early syphilis patient after treatment using penicillin. In this study, we identified the molecular subtypes of *T*. *pallidum* and analyzed potential risk factors associated with serofast status. We also measured chemerin levels in the serum of patients in order to find a clinical serologic marker for the serofast status. We hope these data contribute to the development of guidelines for the management of syphilis patients who experience serofast after treatment.

## Methods

### Study population

This prospective study was conducted in ten major hospitals located in ten prefectural-level cities in Jiangsu Province, China. The Sexually Transmitted Disease (STD) Clinics in these hospitals regularly receive a large number of patients. Between August 2011 and July 2015, eligible patients visiting the STD Clinics were referred to participate in this study. All patients were asked to complete a questionnaire regarding demographic information and to sign an informed consent form. The study design was approved by the ethics committee of Institute of Dermatology, Chinese Academy of Medical Sciences.

### Diagnosis criteria and treatment

According to CDC guidelines in China, primary syphilis presented genital ulcers (chancre) with or without regional lymphadenopathy, secondary syphilis manifested a maculopapular rash in palmar /plantar or condylomata. Dark field examination was used to detect the presence of *T*. *pallidum* in moist lesions (e.g. chancre or condylomata). A reactive nontreponemal test and a treponemal test were used to confirm the diagnosis of primary and secondary syphilis. In order to identify molecular subtypes of *T*. *pallidum*, only patients with moist lesions and a negative HIV test were enrolled in this study. Non–penicillin-allergic patients received initial treatment with benzathine penicillin (2.4 million units by intramuscular injection, once weekly for 3 weeks, 7.2 million units in total). Penicillin-allergic patients received doxycycline (100 mg taken orally, twice daily for 15 days). At baseline and 3, 6, 9 and 12 months after treatment, the rapid plasma reagin (RPR) test was carried out on patient sera.

Six months after treatment, serological response was defined as either a negative RPR titer or more than a fourfold (two dilutions) decline in titers, and serological cure was defined as a negative RPR titer at 12 months [18]. Serofast status was defined as either no change in RPR test results or less than a twofold (one dilution) decline or increase in titers at 12 months, as compared with baseline titers [19]. All participants who had less than a fourfold decrease in titers after treatment received retreatment and were tested for HIV again.

### Collection of clinical specimens

Venous blood (5 mL) was draw from all patients and healthy donors for HIV and syphilis tests. Exudates of moist lesions were transferred to storage medium and kept at -70°C until they could be transported to the Institute of Dermatology, Chinese Academy of Medical Sciences, for molecular subtyping.

The RPR test and the *T*. *pallidum* particle agglutination (TPPA) test were used to confirm diagnoses of syphilis. The primary anti-HIV antibody screening was conducted using an enzyme-linked immunosorbent assay (ELISA) (Wansheng Biotech Inc., Beijing, China). A western blot assay (HIV Blot 2.2, Genelabs Diagnostics, Singapore) was used to confirm the presence of anti-HIV antibodies.

### Molecular subtyping of *T*. *pallidum*

QIAamp DNA minikit (Qiagen) was used to extract specimens’ DNA, and the *tpp47* gene of *T*. *pallidum* was amplified by using PCR assay. The amplified conduction with *tpp47* gene positive was stored at -70°C for detecting molecular subtypes of *T*. *pallidum*.

As previously described by Marra et al. [20], the size of the *arp* gene, banding pattern of the *tpr* gene, and sequence of the *tp0548* gene were integrally analyzed to identify *T*. *pallidum* subtypes. Other than *tpr* gene, which was amplified with a nested PCR assay, the *arp* gene and the *tp0548* gene were amplified using standard PCR assay. Primer sequences of the three genes were shown in Table 1. The amplification conditions for *arp* were 94°C for 5 minutes, 40 cycles of 94°C for 1 minute, 60°C for 1 minute, and 72°C for 1 minute, followed by a final extension step of 72°C for 8 minutes. The *arp* amplicons were electrophoresed and then analyzed to determine their sizes. The amplification conditions for *tpr* were the same as those for the *arp* gene, except that the annealing was performed at 61°C in the second round. The *tpr* amplicons were digested with the restriction endonuclease MseI, and the resulting digestion patterns were compared with data in the article by Pillay et al. [21]. PCR conditions for *tp0548* were as follows: 95°C for 2 minutes, then 40 cycles of 95°C for 1 minute, 62°C for 1 minute, and 72°C for 1 minute, followed by a final extension step of 72°C for 8 minutes. The production of *tp0548* gene was sequenced for genotyping.

**Table 1:** Primers used in *T*. *pallidum* strain typing.

Primer	Sequence
*tpp47* sense	*5’- CGTGTGGTATCAACTAGTG -3’*
*tpp47* antisense	*5’-TCAACCGTGTACTC GTGC -3’*
*arp* sense	*5’-CAA GTC AGG ACG GAC TGT CCC TTG C-3’*
*arp* antisense	*5’-GGT ATC ACC TGG GGA TGC GCA CG-3’*
*tpr sense 1*	*5’-ACT GGC TCT GCC ACA CTT GA-3’*
*tpr antisense 1*	*5’-CTA CCA GGA GAG GGT GAC GC-3’*
*tpr sense 2*	*5’-CAG GTT TTG CCG TTA AGC-3’*
*tpr antisense 2*	*5’-AAT CAA GGG AGA ATA CCG TC-3’*
*tp0458 sense*	*5’-GGT CCC TAT GAT ATC GTG TTC G-3’*
*tp0458 antisense*	*5’-GTC ATG GAT CTG CGA GTG G-3’*

Note: The primers *tpr* sense 1 and *tpr* antisense 1 were used in first PCR round. The primers *tpr* sense 2 and *tpr* antisense 2 were used in second PCR round.

### Measurement of chemerin

An ELISA assay was used to determine the chemerin serum levels using Human RARRES2 ELISA Kit (Sigma-Aldrich, St. Louis, MO, USA), following the manufacturer’s instructions. Briefly, 100 μl serum sample was added into wells and incubated for 2.5 hours, and then were incubated for 1 h with biotinylated detection antibody. The assay was developed by addition of TMB reagent and was stopped by addition of stop solution. Final data were read at a wavelength of 450 nm. The mean absorbance was calculated for each set of duplicate standards, controls, and samples. The concentration of sample was accounted according to the standard curve.

### 2.6. Statistical analysis

We used SPSS 20.0 software (SPSS Inc., Chicago, IL) to conduct all statistical analyses. Chi-square and Fisher’s exact tests were conducted to analyze the relationship between clinical characteristics and serofast state, as well as the relationship between molecular subtypes and serofast state. Logistic regression analysis was used to detect associated factors with the serological outcome after treatment. The significance of chemerin levels and other parameters was assessed using unpaired t-tests. P-values less than 0.05 were used to consider statistical significance for all analyses.

## Results

### Clinical characteristics

**Fig 1:**
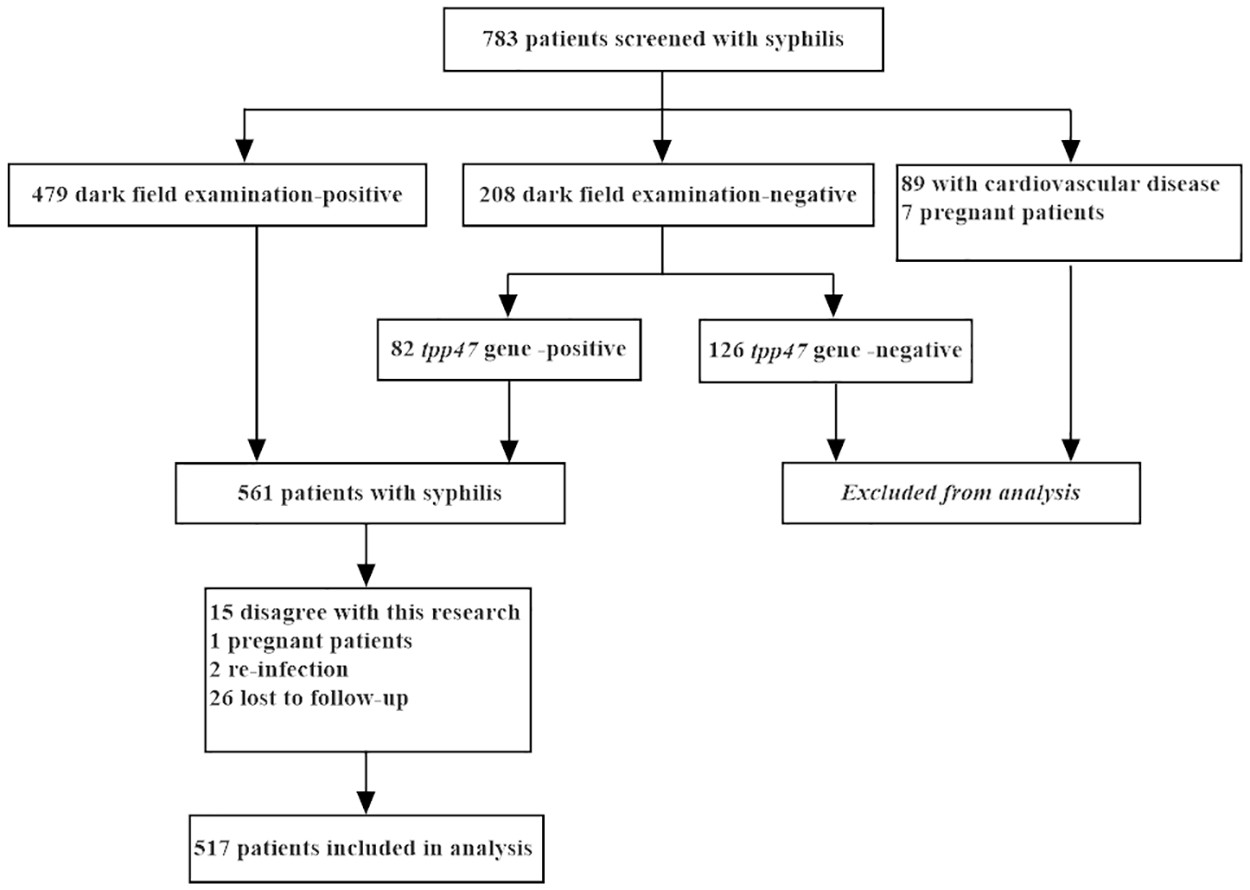
Patients selection flowchart.

From August 2011 and July 2015, a total of 783 patients were identified, 517 of whom were enrolled in this study (Fig 1). Nearly two-thirds (56.9%) of the patients were married, and 73.2% were male. Of the 517 patients, 401 had primary syphilis and 116 had secondary syphilis. At baseline, 64 patients were HIV positive, while 31 patients exhibited HIV seroconversion at 12 months after treatment, resulting in a HIV prevalence of 18.4%. Among the 517 patients, 161 reported that they had had sex with man in the past 6 months.

There were 452 non-penicillin-allergic patients who received benzathine penicillin and 65 penicillin-allergic patients who received doxycycline. Twelve months after treatment, 79.3% (410/517) of the patients achieved serological cure, 20.1% (104/517) were serofast, but 0.6% (3/517) were serological failures. For these three patients, there was no evidence to explain their treatment failure. Eighty-three percent (339/410) of the serologically cured patients had primary syphilis. By contrast, only 59.6%(62/104) of the patients exhibiting serofast had primary syphilis. Compared to the serological cure patients, patients with serofast status were older. The proportion of patients with a baseline titer ≤1:8 was higher for patients showing serofast than for those who were cured (Table 2).

**Table 2:** Comparison of characteristics between serological cure and serofast status.

	Serofast Status (n = 104)	Serological Cure (n = 410)		
Characteristic	n (%)	n (%)	Adjusted χ^2^	*P* value
Age(yr)			103.3	<0.01*
<20	4(3.8)	62(15.1)		
20–40	27(26.0)	269(65.6)		
>40	73(70.2)	79(19.3)		
Gender			1.3	>0.05^NS^
Male	84(80.8)	309(75.4)		
Female	20(19.2)	101(24.6)		
Sexual orientation			0.5	>0.05 ^NS^
Homosexual	29(27.9)	68(16.6)	5.5	
Bisexual/heterosexual	75(72.1)	342(83.4)		
Anal sex with men in the past 6 months			2.7	>0.05 ^NS^
Yes	40(38.5)	121(29.5)		
No	64(61.5)	289(70.5)		
HIV-positive			3.4	>0.05 ^NS^
Yes	19(18.3)	45(11.0)		
No	85(81.7)	365(89.0)		
Syphilis stage			24.4	<0.01*
Primary	62(59.6)	339(82.7)		
Secondary	42(40.4)	71(17.3)		
Baseline RPR titer			26.0	<0.01*
≤1:8	60(57.7)	127(31.0)		
1:16–1:32	26(25.0)	197(48.0)		
≥1:64	18(17.3)	86(21.0)		

Abbreviations: RPR, rapid plasma regain; *for significance; NS for non-significance; Chi-square test was conducted; *P*-values less than 0.05

### Molecular subtypes

**Fig 2:**
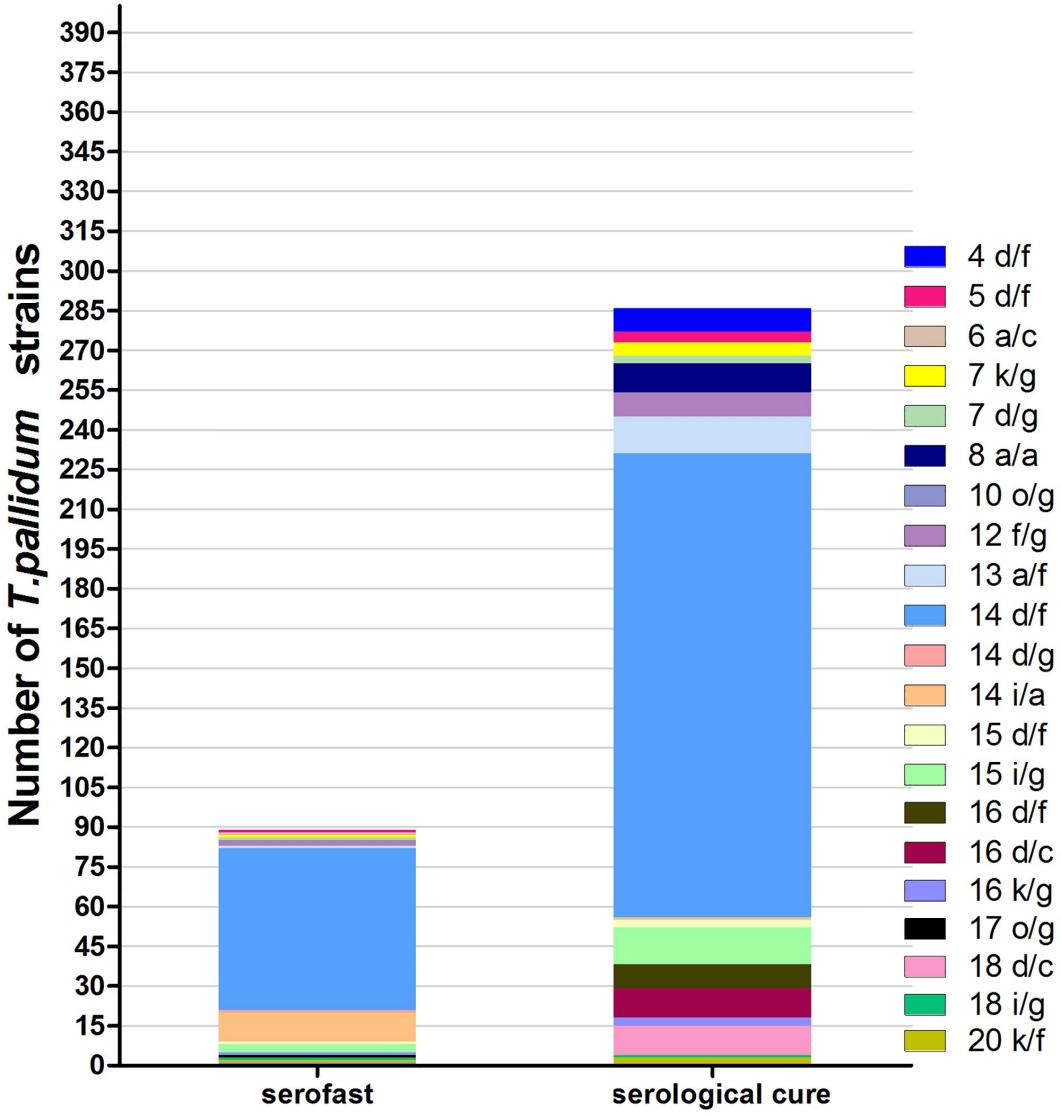
Distribution of *T*. *pallidum* strains isolated from serofast patients and serological cure patients, 2011 to 2015. The 14d/f type was the predominant subtype in two groups patients. The novel type of 14 i/a might predict an increasing risk of serofast status (χ^2^ = 34.4, *P* < 0.01).

In the present study, one aim was to examine some *T*. *pallidum* strains which might associated with serofast status of early syphilis patients. Overall, we obtained 462 samples containing *T*. *pallidum* DNA, 416 of which shown the presence of *tpp47* gene. Only 375 specimens full strain subtypes were identified and analyzed in the study, 104 of 375 specimens were isolated from serofast patients and 286 from serological cure patients. A total of fourteen *arp* repeat sizes (4, 5, 6, 7, 8, 10, 12, 13, 14, 15, 16, 17, 18 and 20 repeats) and five *tpr* RFLP patterns (a, d, i, k and o) were detected. The most common *arp* type was 14, and the most common *tpr* type was d. We identified four *tp0548* sequence types (a, c, f, and g). Combining the three genes types yielded 21 strain types. The 14d/f type was the predominant subtype (58.6% in patients experiencing serofast and 61.2% in cured patients, χ^2^ = 0.21, P > 0.05). Eleven (10.6%)of 104 patients with serofast status were infected with subtype 14i/a. The proportion was significantly higher than that of patients with serological cure (χ^2^ = 34.4, P < 0.01). The novel type of 14i/a might predict an increasing risk of serofast status (Fig 2).

### Serum levels of chemerin

**Fig 3:**
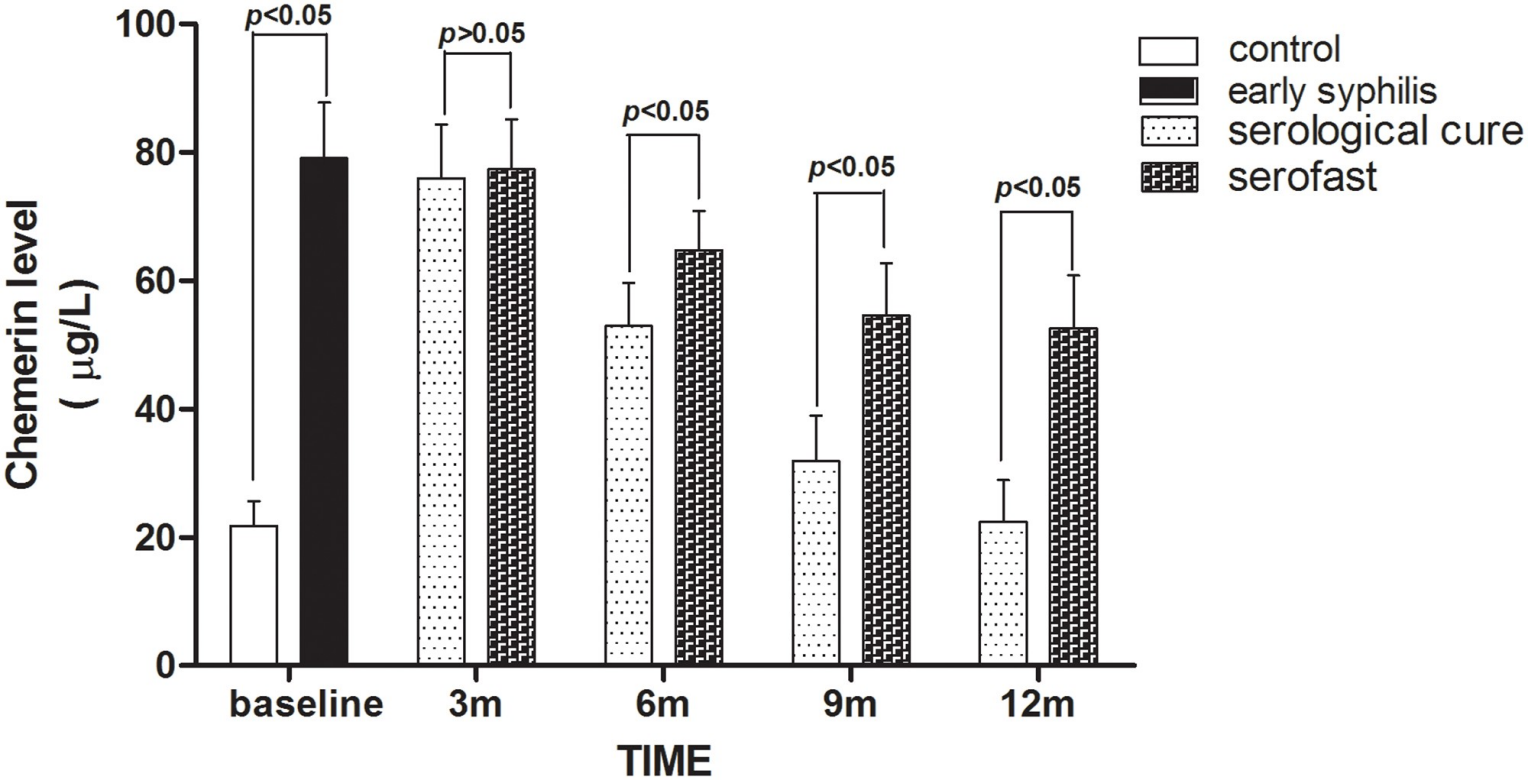
Serum levels of chemerin of early syphilis patient. Detection serum levels of chemerin in control group (n = 30), early syphilis patients (n = 517), serological cure patients (n = 410) and serofast status patients (n = 104). The concentrations of chemerin were determined using a standard curve. Data are shown as the serum concentration of chemerin, and each bar represents the mean ± standard error. Unpaired t-tests was used to assess the significance of chemerin levels. The control group are healthy uninfected patients.

Chemerin is an adipocytokine that has a strong chemotactic effect on dendritic cells and macrophages and plays an important role in the early inflammatory response. We previously found chemerin levels was higher in early syphilis lesions than in healthy tissue (data is included in another manuscript that has been submitted but not published). In this study, we found that chemerin levels at baseline were significantly higher in early syphilis patients than in healthy uninfected patients (79.1 μg/L versus 21.8 μg/L, p<0.01). Subsequently, we compared chemerin levels between serological cure patients and serofast patients at 3-, 6-, and 9-month time points after treatment. Chemerin levels decreased at 3-month in two groups (76.0 μg/L in serofast group versus 77.4 μg/L in serological cure group, p>0.05), and continued to decrease at 6-month (52.9 μg/L versus 64.8 μg/L, p<0.01). Twelve months after treatment, chemerin levels in serological cure patients dropped to 22.4μg/L, more than 2.0-fold lower than those in serofast patients (52.5μg/L, p<0.01) (Fig 3).

### Serological responses of serofast patients

**Fig 4:**
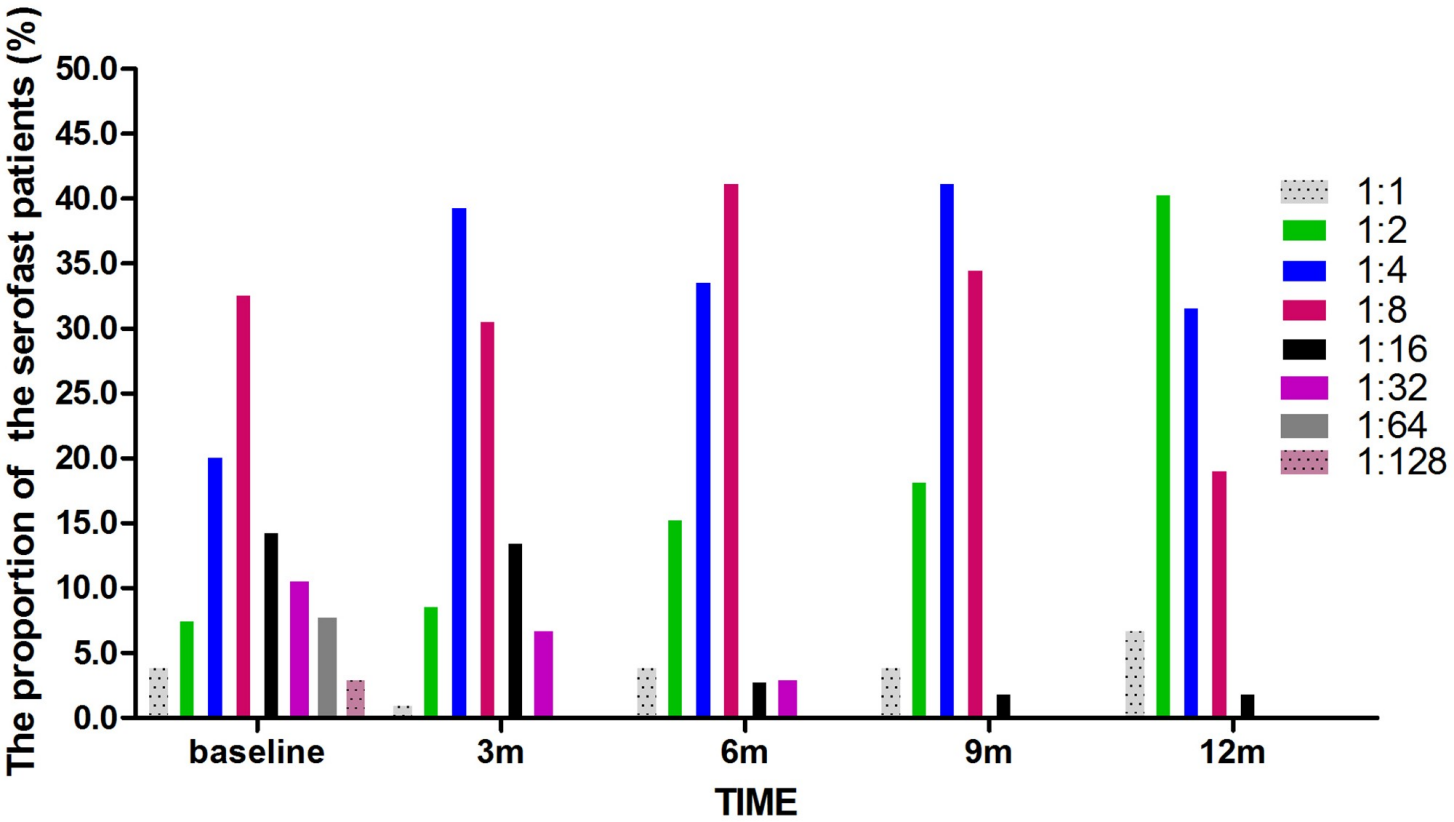
The proportion of serofast patients by different time points RPR titre. At different time points, the RPR titres in serofast patient changed. The proportion of patients with RPR titres of ≤1:8 increased from 64.4% at baseline to 98.0% at 12 months.

Among 104 patients with serofast state, the RPR titres in serofast patient varied at different time points. The major RPR titre in serofast patients was 1:8 (32.7%) at baseline time, however, the RPR titre of 1:2 became dominant (40.4%) at 12 months. The proportion of patients with RPR titres of ≤1:8 increased from 64.4% at baseline to 98.0% at 12 months (Fig 4) (Table 2).

### Factors associated with serofast

Risk factors potentially associated with serofast status were evaluated by logistic regression. In the multivariate analysis, age was a risk factor of serofast status. Older syphilis patients (>40 years) after treatment were more likely to exhibit serofast status. Another risk factor was baseline RPR titer. Syphilis patients with lower baseline RPR titer (≤1:8) might predict a higher risk of serofast status (Table 3).

**Table 3:** Multivariate analysis of factors associated with serofast patients.

	multivariate		
Characteristic	n^#^	OR (95% CI)	*P* value
Age(yr)			
<20	4(63)	1	
20–40	27(271)		
>40	73(79)	5.75(4.27–8.84)	<0.01*
Syphilis stage			
Primary	62(339)	1	
Secondary	42(74)	1.32(0.79–1.79)	0.08 ^NS^
Baseline RPR titer			
≤1:8	60(129)	2.03(1.83–4.01)	<0.01*
1:16–1:32	26(197)		
≥1:64	18(87)	1	

Abbreviations: OR, odds ratios; RPR, rapid plasma regain; *for significance; NS for non-significance; Logistic regression analysis was used; *P*-values less than 0.05.

^#^ serofast status (serological cure)

## Discussion

Even though it is challenging to interpret the relationship between a syphilis patient outcome and the results of a nontreponemal antibody test, the test is still the most popular method for monitoring and evaluating therapeutic responses [22–23]. This may be because of a lack of more sensitive and specific methods to detect *T*. *pallidum* [24–25]. The substantial proportion of serofast status patients was about 12.0% at 12 months after treatment worldwide [26]. In present study, 20.1% of patients with early syphilis exhibited serofast status. This number was higher than that reported by some studies [27–30], but similar to data from elsewhere in China that were reported by Tong et al. [31].

Given the proportions of patients with serofast status was high, it has interested a range of investigators around the world to study its associated risk factors, in order to find the predictor for managing patients with serofast. Previous investigators suggested that the patient’s immune system and the treatment regimens could affect the serological response after treatment [26], as well as the subtypes of *T*. *pallidum* and neurosyphilis and HIV infection [15, 31]. It also has been hypothesized that the persistent infection and antigenic variation in *T*. *pallidum* resulted in the serofast condition [19]. However, few studies served as proof of this hypothesized to this day.

A previous study speculated that the differences in strains’ pathogenecity might contribute to the risk of serofast status [31]. In order to further investigate the association between *T*. *pallidum* strains and serofast status, we identified the *T*. *pallidum* molecular subtypes infecting early syphilis patients. The 14 d/f was the predominant subtype in all patients, including serofast patients. Interestingly, we identified a novel subtype, 14i/a, which has not been reported in previous studies. Statistical analysis showed that the proportion of 14i/a type in serofast patients was significantly higher than that in patients with serological cure. The i subtype of *tpr* gene in *T*. *pallidum* has been considered as an important predictor of serofast in a previous research [15,32]. The novel type of 14i/a might predict an increasing risk of serofast status. Further convincing evidences from clinical patients and animals were needed to investigate the pathogenecity of the 14i/a strain and the relationship with serofast status.

The clinical and serological characteristics associated with therapy response are important concern of clinicians and patients. Therefore, previous research argued the relationship between the clinical characteristics and serological response, expecting to establish recommendation for clinicians to identify and manage syphilis patients with serofast status [31,33]. A recent study reviewed the risk factors of clinical characteristics associated with serofast status. It suggested that older age, gender, infection stage, baseline RPR titer, and cellular immune suppression should be considered as important predictors of serofast [15]. In accordance with these results, older age (>40 years), in our study, was also associated with serofast status. As well as previous study reported by Tong et al., patients >40 years old were more likely to achieve a serofast state than patients < 23 years old (*P*<0.05). The main reason may be because that, in older-aged patients, the immune system was senescent and the humoral response during *T*. *pallidum* infection was weaker, which would influence the serological outcome [34].

Lower baseline RPR titer was a risk factor for serofast status that had been frequently studied [31]. Our results demonstrated that early syphilis patients with baseline RPR titer of ≤1:8 had significantly higher probability of exhibiting serofast status than patients with baseline RPR titer of > 1:8 (*P*<0.05). Lower baseline RPR titer may indicate that the immune system was suppressed or malfunctioning, and thus was not able to mount an efficient inflammatory and immune response, which might due to the failure of rapid and thorough clearance of the spirochete. However, other investigators recently found that the proportions of immune cell types in serofast patients did not differ from those in healthy people [35]. By contrast, Tong et al reported that patients with baseline RPR titres of 1:1 were more likely to achieve serological cure as well as patients with baseline RPR titres of ≥1:32 [31]. Considering that only 11 patients with baseline RPR titres of 1:1 were included into this present study, we did not analyze the proportion of patients showing serofast at 12 months. Additional investigations focused on immune system will help to unravel the immunity mechanism underlying serofast status.

Chemerin is a recently characterized multifunctional protein that plays an important role in many inflammatory conditions and metabolic disorders [36–38]. Chemerin serves as a chemoattractant during early inflammatory responses and involves in the recruitment of dendritic cells, macrophages, and NK cells [39–42]. However, during later stages of inflammation, chemerin could induce the expression of anti-inflammatory cytokines and the generation of anti-inflammatory lymphocytes [43]. Chemerin also works as a vasoactive adipokine that affect the molecular and cellular processes of endothelial cells, which result in vascular injury and dysfunction [44–45].

In our previous study, the results demonstrated that chemerin was highly expressed in human brain microvascular endothelial cells co-cultured with T. pallidum and in lesions of early syphilis (data were included in another manuscript that has been submitted but not published). We speculated that chemerin might play a role in the inflammation response during the pathogenesis of syphilis. Our data here showed that the baseline serum level of chemerin was high in early syphilis patients. At 6 months after treatment, the chemerin level in serofast patients, decreasing in response to treatment, was still higher in serofast patients than in serological cure patients and remained higher in serofast patients at 12 months after treatment (Fig 3), which further confirmed our hypothesis. Did higher chemerin level indicate serofast state? Could we predict serofast at 6 months after treatment according to chemerin level? Much more amount of clinical data is required to answer those questions. Previous researches have reported that, compared with healthy group and serological cure group, the serum levels of interleukin 2 (IL-2) and IL-6 were increased in serofast patients [46–47]. The relationship between the two interleukins and chemerin has not been investigated. Furthermore, it is also unknown that whether the 14i/a subtype of T. pallidum strain induced the expression of chemerin and whether this is a result of persistent spirochetes in the patient’s body. To address these questions, further research is necessary.

There are several limitations to this study, and therefore the results should be interpreted with caution. First of all, we excluded early latent syphilis patients from our analysis of molecular subtypes, which might have affected the statistical analysis of serological data. In addition, we had no standard definition for serofast status, and it is unclear whether 12 months is the optimal time point for assessing serological responses. Finally, the specimens were obtained only from patients in the Jiangsu province of China and the sample size was limited. More research in other regions using larger samples would provide more statistical power.

## Conclusion

We conducted a prospective study on serofast status of syphilis patient. Our data suggested that, after treatment, older age (>40 years) and lower baseline RPR titer (≤1:8) were associated with serofast status in patients with early syphilis. We also identified 21 *T*. *pallidum* molecular subtypes infecting early syphilis patients and detected a new subtype, 14i/a. We found that the proportion of 14i/a type in serofast patients was significantly higher than in patients with serological cure, predicting an increasing risk of serofast status. Our results indicated that the serum level of chemerin in serofast patients was higher than that in serological cure patients, suggesting that may be a novel cytokine marker of serofast outcome in early syphilis patients after therapy.
